# Superparamagnetic Properties of Hemozoin

**DOI:** 10.1038/srep26212

**Published:** 2016-05-18

**Authors:** M. Inyushin, Yu. Kucheryavih, L. Kucheryavih, L. Rojas, I. Khmelinskii, V. Makarov

**Affiliations:** 1 Universidad Central del Caribe, Bayamón, PR 00960-6032, USA; 2 University of the Algarve, FCT, DQB and CIQA, 8005-139, Faro, Portugal; 3 University of Puerto Rico, Rio Piedras Campus, PO Box 23343, San Juan, PR 00931-3343, USA

## Abstract

We report that hemozoin nanocrystals demonstrate superparamagnetic properties, with direct measurements of the synthetic hemozoin magnetization. The results show that the magnetic permeability constant varies from μ = 4585 (at −20 °C) to 3843 (+20 °C), with the values corresponding to a superparamagnetic system. Similar results were obtained from the analysis of the diffusion separation of natural hemozoin nanocrystals in the magnetic field gradient, with μ = 6783 exceeding the value obtained in direct measurements by the factor of 1.8. This difference is interpreted in terms of structural differences between the synthetic and natural hemozoin. The *ab initio* analysis of the hemozoin elementary cell showed that the Fe^3+^ ion is in the high-spin state (*S* = 5/2), while the exchange interaction between Fe^3+^ electron-spin states was much stronger than *k*_*B*_*T* at room temperature. Thus, the spin dynamics of the neighboring Fe^3+^ ions are strongly correlated, lending support to the superparamagnetism.

Malaria is caused by *Plasmodium spp*., parasitic single-celled protozoan, transferred to humans by mosquitoes. After the mosquito bite, the infection spreads to the liver and next to the blood, where the plasmodium multiplies asexually inside erythrocytes, feeding on their content and destroying the cells. Then parasites spread from one red cell to another, usually synchronously, resulting in further infection and producing attacks of shivering fever every 24–48 hours.

Because hemoglobin is the main protein in the red blood cells, the asexual forms of *Plasmodium spp.* are feeding by degrading the protein part of it, and producing free heme moieties as a byproduct. Being very toxic, the heme must be neutralized by the parasite. The heme is converted in the digestive vacuole of the parasite (at pH about 5) into an insoluble “malaria pigment” hemozoin, that is essentially a heme polymer. The formation of hemozoin is apparently the primary mechanism of heme detoxification in malaria parasites^1^. A different view is that only some 30% of the heme is converted to hemozoin, while the main neutralization occurs via direct degradation of heme with accumulation of iron in the parasite^2^. The heme neutralization process is one of the main targets of the antimalarial drugs, with different researchers expressing different views on whether the drugs affect catalytic enzymes or direct crystallization of hemozoin, or both, or alternatively affect the direct oxidation of heme^1^,^2^,^3^,^4^,^5^,^6^,^7^,^8^,^9^,^10^.

Still, all of the authors agree that hemozoin is present in the digestive vacuole of all species of the malaria parasites. Hemozoin crystals have brick-like shape (1:1:8) with their maximum dimension at 50–1000 nm, depending on the species of *Plasmodium*^6^,^11^,^12^. The structure of hemozoin, according to infrared spectra and X-ray diffraction patterns, is similar to that of ß-hematin, with hemozoin probably resulting from ß-hematin dimer polymerization^13^,^14^. The artificial β-hematin polymer crystals synthesized from hemin chloride under acidic conditions are chemically identical to the hemozoin crystals of biological origin, although having different shape and size, ranging from 50 nm to 20 μm^12^. Interestingly, β-hematin crystals in aqueous solutions tend to aggregate even within minutes of vigorous sonication^12^, which we suggest may occur due to their magnetic properties.

More than a half a century ago it was discovered that erythrocytes with parasites may be separated from the uninfected cells using permanent magnets^15^, and this method was refined in subsequent studies^16^,^17^. It was shown that hemozoin itself may be separated in a similar way^17^,^18^, and that hemozoin content inside the infected cells are the source of driving force that determine the infected cells magnetophoretic mobility^19^. But, based on the famous early work of Pauling^20^,^21^ that determined two possible spin configurations of electrons in the heme iron as diamagnetic or paramagnetic, depending on its oxygenated or deoxygenated form, the hemozoin magnetic properties were considered paramagnetic in the majority of the recent studies that discussed its magnetic properties.

The difference in the magnetic permeability between ferromagnetics and paramagnetics is well known, with paramagnetics having very low permeabilities. Recently it became clear that the magnetic properties also depend on the particle size. In fact, magnetization may randomly flip its direction due to thermal motion in sufficiently small nanoparticles of ferromagnetic materials. Thus their average magnetization is zero in absence of an external magnetic field, therefore they are said to be in the superparamagnetic state. An external magnetic field can magnetize the nanoparticles, same as if they were paramagnetic. However, their magnetic susceptibility is much larger than that of paramagnetic materials.

Here we present direct magnetic permeability measurements of hemozoin crystals, using the method of sample magnetization measurements. We also present calculations showing that paramagnetic hemozoin particles could not produce the observed values of the magnetophoretic mobility in liquid mixtures. Indeed, the energy of Zeeman splitting in paramagnetic systems for the states with nonzero electronic angular momentum is much smaller than *k*_*B*_*T* in reasonable magnetic fields, here *k*_*B*_ is the Boltzmann constant and *T* the absolute temperature. It was found earlier^22^,^23^ that hemozoin has paramagnetic Fe^3+^ centers in high-spin configuration (*S* = 5/2). However, this paramagnetism can’t explain the observed magnetization of hemozoin. Presently we report direct measurements of hemozoin magnetization and the detailed theoretical analysis of its magnetic properties. We find that submicrocrystals and nanocrystals of hemozoin demonstrate superparamagnetic properties; therefore, the exchange interaction between the neighboring iron ions within the crystals is significantly larger than *k*_*B*_*T*. The hemozoin superparamagnetism is also explored using *ab initio* calculations.

## Experimental Methods and Materials

### Materials

Commercial hemozoin (InvivoGen, France; 93–95%) was used as obtained in the direct measurements of hemozoin magnetization. The average crystal size was 200–300 nm; no information on crystal structure was available from the vendor.

### Experimental Setup

Magnetic properties were measured using a 7400 series vibrating sample magnetometer (VSM) from Lake Shore Cryotronics Inc. (2T maximum magnetic field; 3” pole gap, 84 Hz sample vibration frequency). We used the external magnetic field range from −1.5 to + 1.5 T. The sample temperature may be set between −154 °C and 254 °C. Digital signal recording provides for averaging the signal over multiple field cycles.

## Experimental Results and Data Analysis

### Experimental data

**Figure 1:**
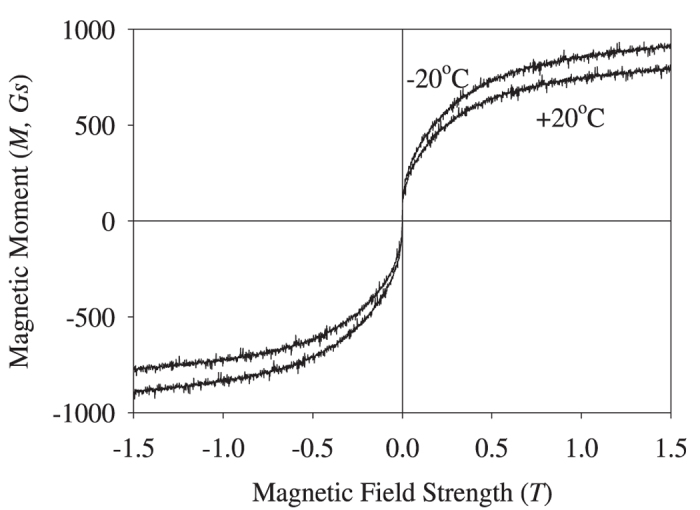
Hemozoin magnetization measured at −20 °C and +20 °C.

The magnetization curve of the 5 mg hemozoin sample was recorded at two temperatures, −20 °C and +20 °C, with the results shown in Fig. 1.

**Figure 2:**
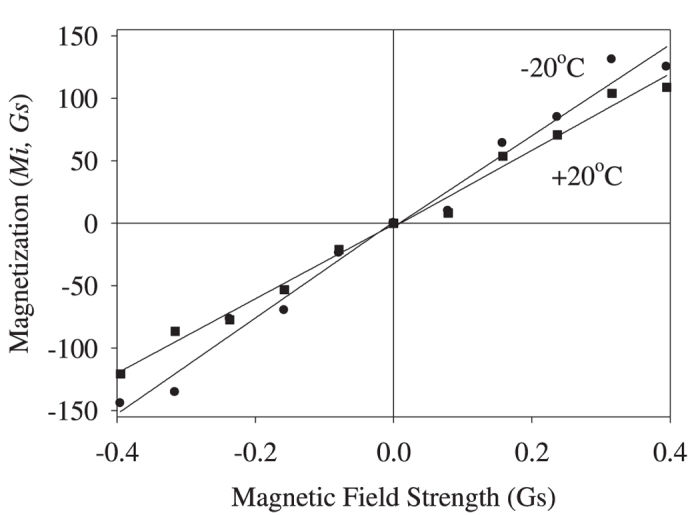
Low-field hemozoin magnetization curves recorded at −20 °C and +20 °C. Note that the external magnetic field values on this scale are comparable to those of the geomagnetic field (0.25 to 0.65 Gs).

The low-field part at the two temperatures is shown in Fig. 2.

The magnetic susceptibility was calculated from the data of Fig. 2, χ_*−*20_ = 365 and χ_20_ = 306 (CGS units). Taking into account that μ = 1 + 4πχ, we thus obtain μ_−20_ = 4585 and μ_20_ = 3843. These values are anomalously large, showing that hemozoin nanocrystals are superparamagnetic. Superparamagnetic properties were reported earlier in such biological systems as red pulp macrophages (cells that destroy damaged erythrocytes by phagocytosis, and somehow neutralize the heme) and ferritin, protein that stores iron^24^,^25^.

We already noted that commercial hemozoin crystals were 200–300 nm in size. However, this value most probably does not refer to single crystals, as the chemically synthesized hemozoin has a broad distribution in crystal size, starting at 10–20 nm. These small crystals easily agglomerate, producing much larger conglomerates^6^,^11^,^12^. We therefore propose, based on the results reported earlier^12^, that such rapid agglomeration may result from magnetic attraction between small hemozoin crystals, which should be significant even in the geomagnetic field (see Fig. 2). Thus, the nanoparticles of the commercial hemozoin should be treated as agglomerates of smaller randomly oriented crystals, as we will do below.

Further on, we shall analyze the quantum state structure in the elementary cell of hemozoin and the role of exchange interactions in model systems, with Fe^3+^ ions interacting via a conjugated π-system. We shall also discuss the experimental data on the magnetic separation of malaria parasites and hemozoin nanocrystals.

### State structure of hemozoin elementary cell (C_4v_ symmetry)

**Figure 3:**
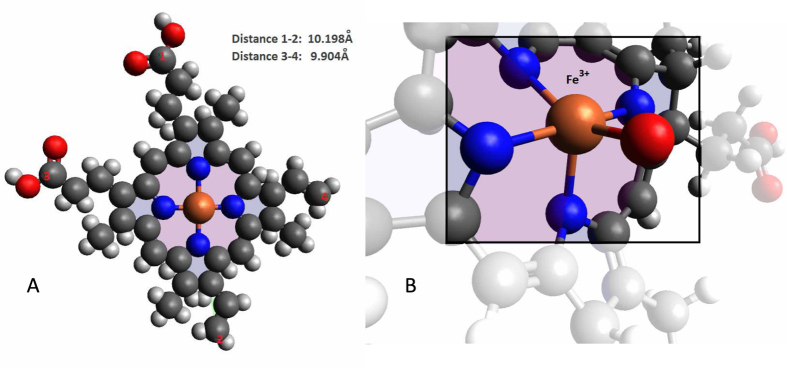
Elementary cell of hemozoin: (**a**) Structure of the parent heme showing the symmetry of the Fe-porphyrine complex and the overall dimensions of the heme; hemozoin is essentially a heme polymer; (**b**) hemozoin elementary cell, showing the Fe^3+^ ion (brown), nitrogen (blue), oxygen (red), and carbon (gray) atoms. Constructed using Version 1.1.1 of the Avogadro open-source molecular builder and visualization tool (http://avogadro.openmolecules.net/) and its import database.

**Figure 4:**
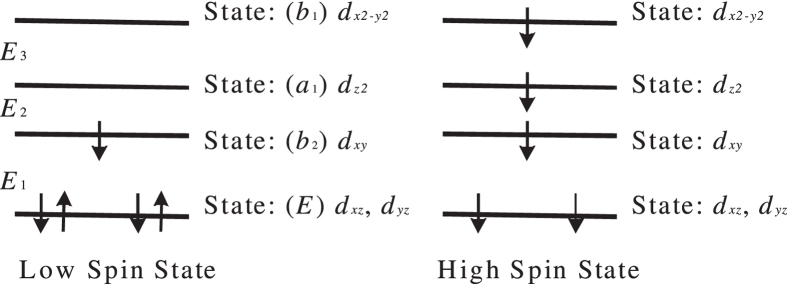
Low- and high-spin states of Fe^3+^ ion in C_4v_ crystal field.

The Fe^3+^ ion is in the high-spin state in hemozoin nanocrystals, as follows from EPR^22^,^23^, magnetophoresis^19^ and magnetization studies^26^. Taking into account the d^5^ electronic configuration of the Fe^3+^ ions in the C_4v_ crystal field of hemoglobin or hemozoin (Fig. 3a,b, respectively), these may exist in either low-spin or high-spin configuration, as shown in Fig. 4.

Figure 3a shows the heme structure in hemoglobin, presenting the symmetry of the Fe-porphyrin-polypeptide complex. The heme diameter is about 11.2 A. Hemozoin is essentially a heme polymer where an additional bond between the two hemes maintains the Fe ion in its Fe^3+^ state, as shown on the simplified structure of the hemozoin elementary cell (Fig. 3b).

The energy *E*_*,i*_ (*i* = 1, 2, 3) of the crystal-field splitting of the *d* states is shown in Fig. 4 as the energy gaps between the neighboring levels. This system has twice-degenerate electronic and vibrational states corresponding to the twice-degenerate irreducible representation (*E*), as shown in Fig. 4, with the local symmetry symbols also shown. Based on the group theory, we analyzed the electronic state structure of the Fe^3+^ ion (see Appendix I), with the electronic ground state being ^2^B_2_ in the low-spin configuration, and ^5^E in the high-spin configuration. In the high-spin state the Jahn–Teller interaction additionally splits the degenerate ground state with a reduction in symmetry, this splitting being much smaller than the crystal field splitting.

The experimental results indicate that the Fe^3+^ ion is in the high-spin configuration^22^,^23^, therefore, the exchange interaction between the d-orbitals of an isolated ion


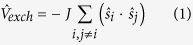


is stronger than the crystal field splitting: 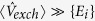. Here, *J* is the electronic exchange integral, and ***s***_***i***_ the electronic spin operator of the *i-*th electron. This result follows from the *ab initio* analysis of the ground-state energies with different total spin for the heme structure shown in Fig. 3, where the globin polypeptide was substituted by an NH_3_ molecule, and all of the free valences in the elementary cell filled by the H atoms. This *ab initio* analysis used Gaussian-2000 commercial software package. The calculations used the coupled-cluster method with the 6–31G (d) basis, for the structure shown in Fig. 3. The calculated energies in function of the total spin are listed in Table 1.

The calculated energies include the exchange interaction of Eq. (1). We see that the lowest-energy state has the maximum spin, with a large gap between the *S* = 5/2 state and the other states. Thus, the ground state should be exclusively populated at room temperature.

### Analysis of the superparamagnetic properties

To understand the superparamagnetic properties of hemozoin nanocrystals, we have to take into account the exchange interaction between the closest Fe^3+^ ions, interacting via π-systems involving carbon and nitrogen atoms:


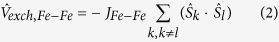


where *J*_*Fe-Fe*_ is the exchange interaction between the closest Fe^3+^ ions, 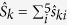, ***s***_*ki*_ is the spin of the *i-*th electron in the *k-*th Fe^3+^ ion. Provided the exchange interaction is stronger than *k*_*B*_*T*, the spins of all of the Fe^3+^ ions in the nanocrystal will be correlated, with the system described by the total spin 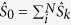, where *N* is the number of iron ions in the nanocrystal. The total spin may vary within the range 5*N*/2, (5*N*/2)-1,…,1/2 or 0, while for the strong exchange interactions the system ground state will have the maximum spin of 5*N*/2 (Hund’s rule). This corresponds to the maximum magnetic moment 
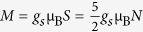
, corresponding to the superparamagnetic properties of the nanocrystal. Here *g*_*s*_ is the electron spin g-factor and μ_B_ is the Bohr magneton.

**Figure 5:**
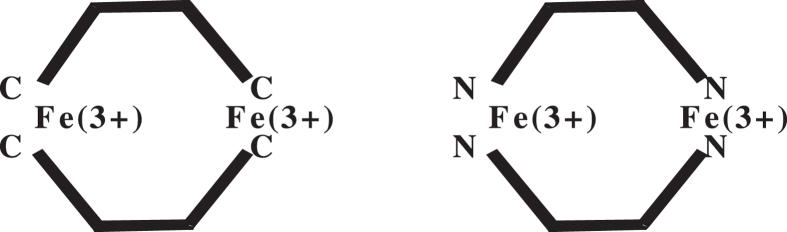
*Ab initio* analysis of the exchange interactions in two cyclic Fe^3+^ complexes Fe_2_(CH_2_ = CH-CH = CH_2_)_2_ and Fe_2_(NH_2_-CH = CH-NH_2_)_2_ was performed using the coupled-cluster method with the 6–31G(d) basis. The energies were calculated for different total spin configurations, with the results listed in Table 2.

We carried out *ab initio* analysis of the two model systems shown schematically in Fig. 5, to provide better understanding of the exchange interactions in the nanocrystals.

It follows from Table 2 that the ground state of the two interacting complexes coincides with the maximum total spin state. Note that the population of the state with *S* = 4 is lower than that of the ground state by the factor of 4 × 10^−6^ and 2 × 10^−5^, respectively. Thus, the magnetic moments of the two model systems coincide with those of their respective ground states.

The results obtained may be extended to hemozoin nanocrystals, where the two closest iron ions are interacting via the respective atomic chains. Once more, the ground state of the hemozoin nanocrystal should have the maximum spin, due to strong exchange interactions between the iron ions.

### Diffusion of hemozoin nanocrystals in aqueous mixtures in the magnetic field gradient

**Figure 6:**
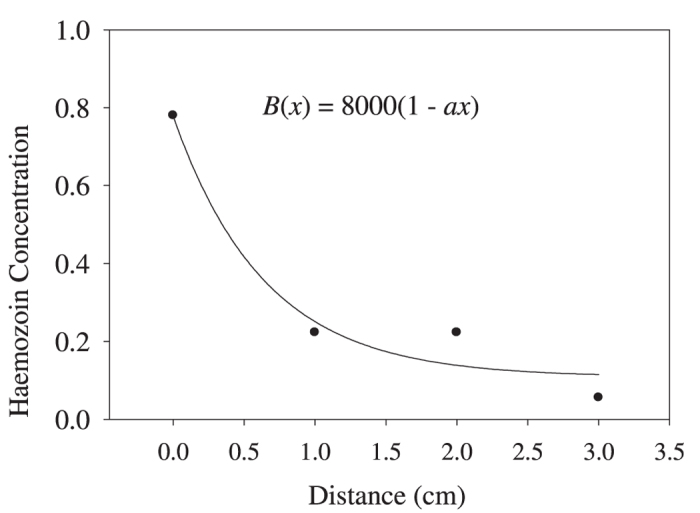
Experimental data from Kim*et al.*^16^ fitted by Eq. (4), the equation gives the magnetic field vs distance from the pole, with *α* = 750 G/cm (to reproduce the conditions used by Kim *et al.*^16^

Hemozoin nanocrystals may be separated by diffusion in a magnetic field gradient (Kim, *et al.*)^16^. Taking into account the magnetic properties of hemozoin nanocrystals, we conclude that strong exchange interactions should exist between Fe^3+^ ions, described by Eq. (2) and creating correlated spin behavior. Thus, the fundamental state of the hemozoin nanocrystal should have the maximum spin *S* = 5*N/*2, *N* being the total number of ions in a crystal. Consequently, hemozoin nanocrystals should be superparamagnetic. Their diffusion in the magnetic field gradient may be described by a one-dimensional equation


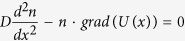


or


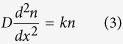


for planar poles and constant magnetic field gradient. The detailed analysis of this equation is performed in Appendix II, with the concentration of nanocrystals given by


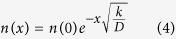


where *D* is the diffusion coefficient in water, *n*(0) is hemozoin nanocrystal concentration in contact with the pole of the magnet (fitting parameter) and *k* is the second fitting parameter. The experimental data by Kim, *et al.*^16^, were plotted as nanocrystal concentration versus distance (see Fig. 6). These data were fitted by the Eq. (4), with the fitted curve also shown in Fig. 6.

The value of the diffusion coefficient was estimated using the relationship (AII.6). The obtained values of the fitting parameters are:


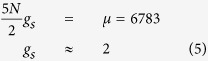


The value obtained here for μ at room temperature is significantly larger than that measured by the direct method, as described in the Experimental data. This difference is apparently due to different morphology of hemozoin particles used presently and by Kim, *et al.*^16^. Namely, we used synthetic hemozoin, while Kim, *et al.*^16^ used natural hemozoin extracted from infected erythrocytes. We expect that synthetic and natural hemozoin have different nanocrystal size distributions, therefore their respective average magnetic moments, proportional to the nanocrystal volume, would be also different. Additionally, hemozoin from different origins may have different impurities, which may also affect its magnetic properties.

## Discussion

Superparamagnetizm was studied earlier by Franken, *et al.*^24^, Gossuin, *et al.*^25^, Tejada, *et al.*^27^ and Jung, *et al.*^28^ for splenic red pulp macrophages, ferric porphyrins and ferritin, respectively. They found μ about 6000 for the biological compounds studied, close to the value of soft iron. Interesting results were obtained for ferritin magnetization^27^, where temperature dependences were investigated. The experimental results were interpreted for ferritin samples in terms of magnetization by macroscopic resonant tunneling^27^,^29^. Note, however, that the magnetic properties of ferritin are determined by nanoparticles of the (FeOOH)_8_(FeOH_2_PO_4_) composition, embedded into the ferritin polypeptide system^30^. The bulk material of this composition is ferromagnetic, while its nanoparticles, smaller than the respective typical magnetic domain, are only superparamagnetic. To compare, Fe_3_O_4_ (magnetite) nanoparticles are also superparamagnetic^31^. Thus, the magnetic properties of ferritin depend on superparamagnetic nanoparticles scattered in the polypeptide matrix, while the magnetic properties of hemozoin depend on the heme polymer, with the closest-neighbor Fe^3+^ ions interacting via the chemical bonds of the respective porphyrine and polypeptide fragments. Thus, the interactions within the ferritin and hemozoin systems are essentially different.

In the present study, direct measurements of the synthetic hemozoin magnetization and indirect evaluation of the natural hemozoin magnetic properties produced μ = 3843 and μ = 6783, respectively, at room temperature. The differences obtained were already discussed. The values obtained show that hemozoin nanocrystals are superparamagnetic.

**Figure 7:**
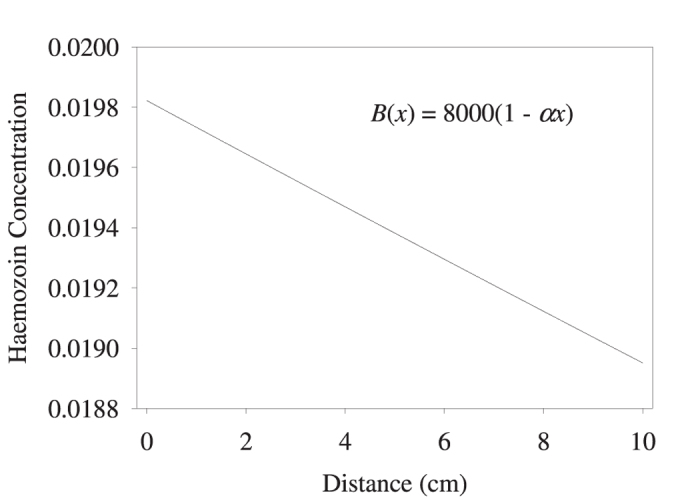
Nanocrystal spatial distribution in the magnetic field gradient of 750 G/cm over the 10 cm distance, calculated assuming paramagnetic nanocrystals. *B*(0) = 8000 G at the pole.

Various studies^1^,^2^,^3^,^4^,^5^,^6^,^7^,^8^,^9^,^10^,^11^,^12^,^13^,^14^,^15^,^16^,^17^,^18^,^19^,^20^,^21^,^22^,^23^ report that hemozoin is paramagnetic, i.e. the exchange interaction between the closest Fe^3+^ neighbors is much smaller than *k*_B_*T*. Thus, all spins of the Fe^3+^ ions should be randomly oriented, with zero total spin of the nanocrystal. We provide a detailed analysis of magnetization of such paramagnetic samples in Appendix III. Using the results of this analysis, we calculated the spatial distribution of paramagnetic hemozoin crystals in the magnetic field gradient, shown in Fig. 7.

We see in Fig. 7 that the concentration difference achieved for paramagnetic hemozoin in these conditions is only 5%, in contrast with the experimental data (reproduced in Fig. 6), where strong concentration changes occur already at 1 cm distance from the pole^16^,^19^,^20^.

The previously obtained value of magnetophoretic mobility of the erythrocyte infected with a late-stage schizont form was *m* = 2.94 × 10^−6^ mm^3^s/kg^19^ corresponding to the net volume magnetic susceptibility relative to water of Δχ = 1.80 × 10^−6^, significantly higher than that of an oxygenated erythrocyte (−0.18 × 10^−6^) but still lower than that of the fully deoxygenated erythrocyte (3.33 × 10^−6^). Taking these results into account, we estimated μ for single non-interacting Fe^3+^ ions in both infected and deoxygenated erythrocytes, using the following relationship for the magnetic susceptibility in a paramagnetic system:


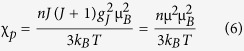


The calculations produced the following results: infected erythrocyte μ = 2.0; deoxygenated erythrocyte μ = 2.6. Here, *n* is the number of the Fe^3+^ ions in the system of interest, and *J* = *S* = 5/2. The estimates are in an acceptable accuracy with the value of 5*g*_*s*_*/*2, which may only be true if the total spin equals 5*n*/2, therefore, all of these systems should be considered superparamagnetic.

We will now discuss the hemozoin EPR data obtained by Sienkiewicz *et al.*^22^,^23^ and the data obtained by Butykai *et al.*^28^. To understand these EPR spectra and interpret the results in terms of the superparamagnetic properties of hemozoin, we need to consider the spin-Hamiltonian for a hemozoin nanocrystal, which may be presented as follows^32^,^33^:


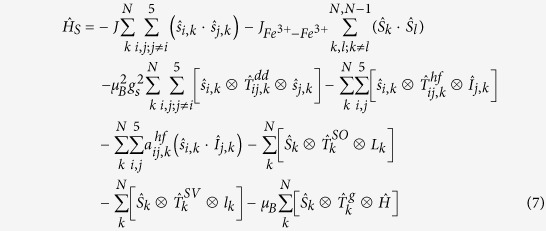


where the first term describes the exchange interactions between the electronic states of the same Fe ion (see above), the second term describes the exchange interactions between the closest Fe^3+^ neighbors (see above), the third term describes the anisotropic spin-spin dipole-dipole interactions, ***T***_*ij,k*_^*dd*^ being the tensor of dipole-dipole interaction, the fourth term describes the anisotropic hyperfine interactions, ***T***_***i**j,k*_^*hf*^ being the tensor of anisotropic hyperfine interaction, *I*_*i,k*_ the nuclear spin of the *i-th* atom interacting with spins of *k-th* Fe^3+^ ion center, *i = k* corresponding to the nuclear spin of the Fe ion considered, the fifth term describes the isotropic hyperfine interactions, *a*_*ij,k*_^*hf*^ being the Fermi contact interaction constant, the sixth term describes the spin-orbit interactions in the frameworks of the Russell-Saunders angular momentum coupling scheme, ***T***_*k*_^*SO*^ being the tensor of spin-orbit interaction of the *k-*th Fe ion, *L*_*k*_ the orbital angular momentum of *k-th* Fe ion, the seventh describes the interactions of the spin angular momentum of Fe ion and vibrational angular momentum of the elementary hemozoin cell, created by anti-phase excitation of the twice-degenerate vibrational modes, ***T***_*k*_^*SV*^ being the tensor of spin-vibrational interaction, ***l***_*k*_ the vibrational angular momentum in the *k-*th hemozoin elementary cell, and the last term is the Zeeman term, ***T***_*k*_^*g*^ being the *g*-factor tensor. Thus, we listed all of the most important terms that define interactions of our system with the external magnetic fields. The first term produces the high-spin configuration with *S* = 5/2 of individual Fe ions; the second term produces the high-spin configuration with *S* = 5*N/*2 of the hemozoin nanocrystal; the terms 3 to 7 define the fine and hyperfine structure of the ground state with *S* = 5*N/*2; the last term is dependent on the magnetic field strength. We shall thus consider the high-field limit, with the last term much larger than the terms 3 to 7. Hence, the spin Hamiltonian may be simplified and presented as:


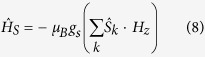


All of the energies will be measured relative to that of the ground state, defined by the first two terms, which are therefore omitted from the Hamiltonian. Thus, the Zeeman splitting energy may be presented as:


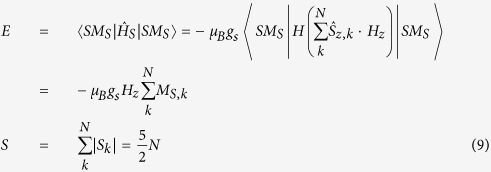


Taking into account the selection rules


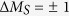


for the magnetic dipole transitions, we may calculate the resonance magnetic field for the EPR in the 3 cm range:


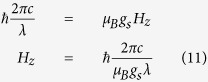


We therefore conclude that the EPR resonance magnetic field strength is determined by the same relationships for both superparamagnetics and normal paramagnetics. Therefore, we conclude that superparamagnetism is not in contradiction with either the earlier reported experimental EPR data^27^,^28^ or the existing theoretical concepts.

In conclusion, we have to note that the analysis of the superparamagnetic properties of hemozoin presented here was carried out for the simple model systems of Fig. 5. We found that the configuration with the maximum total spin (*S* = 5) has the lowest energy. Detailed *ab initio* analysis of hemozoin dimer structure was reported earlier^29^,^30^. However, previous authors did not analyze the effect of the exchange interaction upon the formation of the high-spin configuration. Other studies^34^,^35^ were focused on hemozoin crystal nucleation, by optimizaing the dimer structure. It was shown^29^,^30^ that the optimal dimer structure is controlled by the long-distance dispersion interaction, however, the spin correlation issues were never addressed. Our future study will look into the hemozoin dimer, in order to obtain theoretic foundations for the superparamagnetic properties of hemozoin nanocrystals.

## Conclusions

We report that hemozoin nanocrystals demonstrate superparamagnetic properties. This conclusion is based on direct magnetization measurements of synthetic hemozoin. We measured large values of the magnetic permeability constant, μ = 4585 (−20 °C) and μ = 3843 (+20 °C), corresponding to a superparamagnetic system. We analyzed the previously published experimental data on the separation of hemozoin nanocrystals in the magnetic field gradient, concluding that these natural nanocrystals are super paramagnetic with μ = 6783, a value even larger than that obtained for the synthetic crystals. *Ab initio* analysis of the model systems with two Fe^3+^ ions in the high-spin configuration coupled by two atomic chains with conjugated π-bonds results in the ground state with the total spin *S* = 5, with the excitation energy into other spin states being much larger than *k*_*B*_*T* = 200 cm^−1^ at room temperature. These results were extended to entire hemozoin nanocrystals. We also conclude that superparamagnetism in itself is not in contradiction with the earlier reported experimental data on EPR of hemozoin crystals and the existing theories.

## Additional Information

**How to cite this article**: Inyushin, M. *et al.* Superparamagnetic Properties of Hemozoin. *Sci. Rep.* **6**, 26212; doi: 10.1038/srep26212 (2016).

## Figures and Tables

**Table 1:** *Ab initio* calculated energies of ground state of hemozoin elementary cell.

*S*	5/2	3/2	1/2
*E*, eV	0 eV	0.87 eV	1.42 eV

Note: we assigned the relative energy of 0 eV to the state with the *S* = 5/2 (with the calculated binding energy of -216.0332451267 Hartree).

**Table 2:** Calculated *ab initio* energies of ground states for the Fe_2_(CH_2_ = CH-CH = CH_2_)_2_ and Fe_2_(NH_2_-CH = CH-NH_2_)_2_ complexes.

Fe_2_(CH_2_ = CH-CH = CH_2_)_2_						
*S*	5	4	3	2	1	0
*E*, eV	0	0.31	0.73	1.05	1.38	1.56
Fe_2_(NH_2_-CH = CH-NH_2_)_2_						
*S*	5	4	3	2	1	0
*E*, eV	0	0.27	0.49	0.78	1.09	1.28

Note: we assigned the relative energy of 0 eV to the state with S = 5 (with the calculated binding energy of -116.07894432 Hartree and

-116.18398821 Hartree respectively, for the two model complexes).
